# Gut microbiota in dysmenorrhea: causal evidence from Mendelian randomization and microbial-targeted intervention validation

**DOI:** 10.3389/fmicb.2025.1720643

**Published:** 2026-02-02

**Authors:** Yajie Qin, Rui Ma, Lili Zhang, Xiaotian Yang, Huifang Zhou, Yanping Wang

**Affiliations:** 1 College of Traditional Chinese Medicine, Changchun University of Chinese Medicine, Jilin, China; 2 Department of Gynecology, The Affiliated Hospital to Changchun University of Chinese Medicine, Jilin, China; 3 Department of Gynecology, Affiliated Hospital of Nanjing University of Chinese Medicine, Nanjing, Jiangsu, China

**Keywords:** 16S rDNA, dysmenorrhea, gut microbiota, Mendelian randomization, traditional Chinese medicine

## Abstract

**Background:**

Dysmenorrhea is a prevalent gynecological disorder with multifactorial pathophysiology, including prostaglandin overproduction, inflammation, and pain sensitization. Emerging evidence suggests that gut microbiota may contribute to pain modulation, although causal relationships remain unclear.

**Methods:**

Bidirectional two-sample Mendelian randomization (MR) was performed to investigate causal associations between dysmenorrhea and gut microbiota. Complementary *in vivo* validation was conducted in a primary dysmenorrhea (PDM) rat model treated with ibuprofen or the traditional Chinese medicine Wenjing Zhitong Decoction (WJZTD). The gut microbiota composition was analyzed using 16S rRNA sequencing, and correlations with pain-related parameters were assessed.

**Results:**

Forward MR analyses revealed that genetic liability to dysmenorrhea influenced the abundance of specific gut taxa, notably reducing Lachnospiraceae genera and increasing Erysipelotrichaceae, which was consistent with observed alterations in PDM rats. Reverse MR provided no robust evidence that gut microbiota causally affect dysmenorrhea. In the PDM model, both ibuprofen and WJZTD produced analgesic effects, but induced distinct microbial signatures: ibuprofen increased the presence of *Staphylococcus*, while WJZTD enriched the population of *Bifidobacterium*. Correlation analyses highlighted *Blautia* as a microbiota feature associated with reduced pain, suggesting that modulation of this genus may represent a potential therapeutic strategy.

**Conclusion:**

This study demonstrates that dysmenorrhea causally alters gut microbiota composition. The restoration of *Blautia* and *Bifidobacterium* by WJZTD is associated with pain alleviation, highlighting gut microbiota modulation as a potential strategy for dysmenorrhea management.

## 1 Introduction

Dysmenorrhea, a global gynecological problem characterized by periodic lower abdominal pain (Song et al., 2025), dyspareunia, menstrual irregularities, and menorrhagia (Chapron et al., 2024), affects over 40% of menstruating women and up to 94% of adolescents (Dixon et al., 2024; Tu et al., 2024). Approximately one-third of dysmenorrhea sufferers are absent from work or school, and affected individuals may also face a higher risk of infertility (Bindra et al., 2022; Yang M. et al., 2024). Based on whether documented pelvic pathology is associated, dysmenorrhea is categorized into primary and secondary types (Coco, 1999).

The pathogenesis of dysmenorrhea is complex and multifactorial. In addition to the widely recognized explanation involving the overproduction of prostaglandins (PGs) (Zhang et al., 2025), the pathological progression of dysmenorrhea also includes inflammatory response (Koppolu et al., 2021), pain sensitization (Li et al., 2025), estrogen dominance, progesterone resistance (Bulun et al., 2023), When it comes to treatment, non-steroidal anti-inflammatory drugs (NSAIDs) are commonly recommended to inhibit PGs secretion, while oral contraceptives (OCs) can regulate estrogen and progesterone levels (Ferries-Rowe et al., 2020). Additionally, Traditional Chinese Medicine (TCM) is frequently employed in clinical practice. TCM is recognized for its multi-component, multi-target, and multi-therapeutic effects, which have demonstrated significant therapeutic benefits in previous studies (Yang et al., 2025). However, further exploration of its underlying mechanisms is still necessary.

Emerging evidence demonstrates that gut microbiota dysbiosis is linked with various pain syndromes, including chronic pain and neuropathic pain (Takahashi et al., 2024; Yao et al., 2023). In dysmenorrhea, this association is primarily mediated by pain regulation along the gut-brain axis (Khawar et al., 2023), inflammatory responses (Goudman et al., 2024), and the estrobolome (Qian et al., 2022). Clinical research has revealed significant differences in the composition of gut microbiota between patients with endometriosis and healthy women (Pérez-Prieto et al., 2024). Furthermore, fecal microbiota transplantation (FMT) and probiotic intervention have demonstrated efficacy in regulating microbiota, reducing inflammation, and lesion size in dysmenorrhea animal models (Takahashi et al., 2024). Consequently, gut microbiota reconstruction is emerging as a novel treatment strategy for dysmenorrhea (Xu et al., 2025).

Although suggestive, the observed associations are subject to confounding by diet, lifestyle, and systemic inflammation, precluding a direct causal inference. Mendelian randomization (MR), which employs genetic variants as instrumental variables, offers more reliable causal estimates than traditional observational studies by reducing confounding and reverse causation (Moen et al., 2020). Previous MR analyses have identified causal relationships between gut microbiota and inflammatory cytokines such as IL-6 and TNF-α, which are implicated in the pathophysiology of dysmenorrhea (Liu et al., 2024). However, to date, no study has employed bidirectional MR to clarify the causal relationship between dysmenorrhea and gut microbiota dysbiosis.

Here, we conducted a bidirectional two-sample MR analysis to clarify the causal relationship between dysmenorrhea and gut microbiota. To complement the genetic evidence, we established a primary dysmenorrhea rat model and examined the effects of ibuprofen and the traditional Chinese medicine Wenjing Zhitong Decoction (WJZTD), which is derived from Wenjing Decoction recorded in *Jingui Yaolue* and has long been used for dysmenorrhea treatment, previously validated for its analgesic properties (Qin Y. et al., 2025). By characterizing gut microbiome alterations, assessing microbial restoration after interventions, and performing correlation analysis with functional prediction, we aimed to uncover the distinct mechanisms through which pharmaceutical and herbal therapies exert their effects, with particular emphasis on the multi-target, pathway-driven actions of TCM.

## 2 Materials and methods

### 2.1 Study design

**FIGURE 1:**
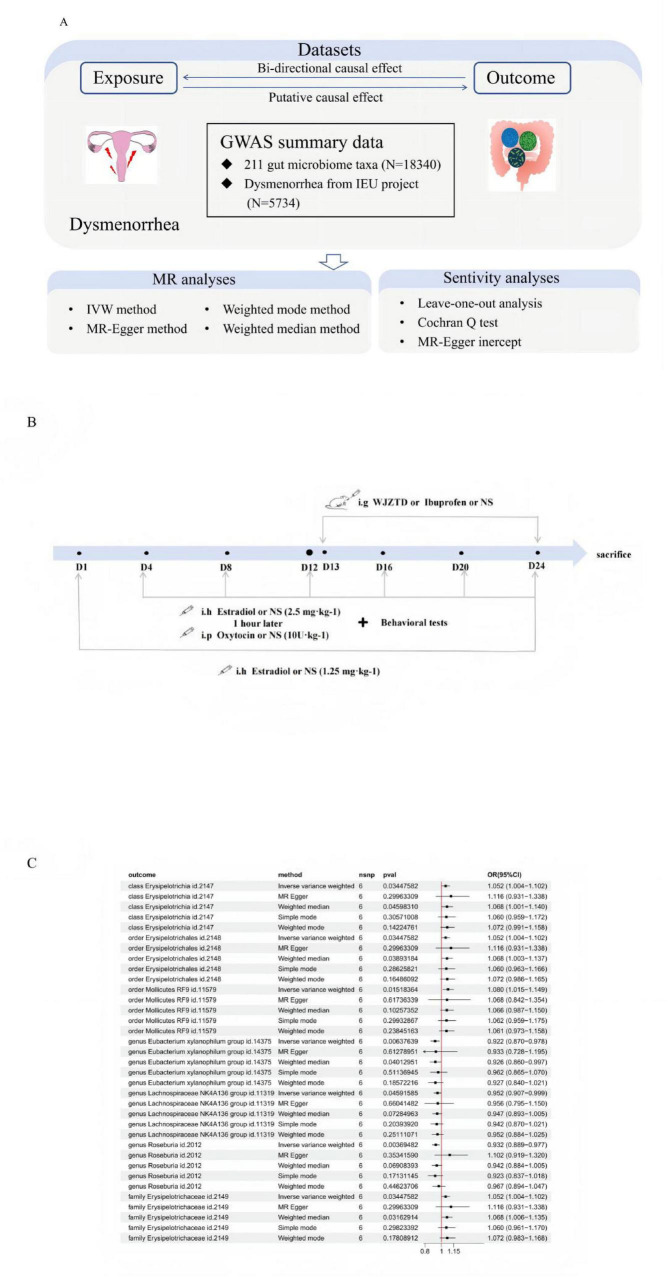
**(A)** Study overview. GWAS, genome-wide association study; IVW, inverse-variance weighted. **(B)** Modeling workflow: Oxytocin was injected on days 4, 8, 12, 16, 20, and 24 to induce dysmenorrhea. Behavioral tests were performed after each episode (*n* = 5), with Wenjing Zhitong Decoction (WJZTD) or ibuprofen administered orally for 12 days after three episodes. **(C)** Mendelian randomization results of the causal effects between gut microbiota and dysmenorrhea based on IVW analysis (*P* < 0.05).

A two-sample MR analysis was performed employing single-nucleotide polymorphisms (SNPs) as instrumental variables(IVs) to assess the potential causal relationship between dysmenorrhea and the human gut microbiome. The study framework is illustrated in Figure 1A.

### 2.2 Source of data

The genome-wide association study (GWAS) summary statistics for dysmenorrhea (5,734 East Asian individuals, ebi-a-GCST006636), obtained from the IEU Open GWAS database^1^, were used as exposures. Outcome data (Supplementary Table 1) on 211 gut microbiota taxa were derived from 18,340 participants in a 2021 study (Kurilshikov et al., 2021). All datasets had prior approval from the relevant ethical review board.

### 2.3 Data filtration and IVs selection

Genetic IVs were selected as genome-wide significant SNPs (forward MR: *P* < 5 × 10^–8^; reverse MR: *P* < 1 × 10^–5^). Standard quality control procedures were applied, which included excluding variants in the major histocompatibility complex (MHC) region, palindromic SNPs, and those with a minor allele frequency (MAF) of <1%. Independent SNPs were identified through PLINK clumping using a linkage disequilibrium (LD) threshold of r^2^ < 0.001 within a 10,000 kb window. To ensure robustness and mitigate weak instrument bias, only instruments with an F-statistic > 10 were retained. All analyses were performed under the three core assumptions of MR.

### 2.4 Bi-directional two-sample MR analyses

#### 2.4.1 Primary analysis

Bidirectional two-sample MR analyses were carried out with the TwoSampleMR R package (Hemani et al., 2018). The primary causal estimates were obtained with the inverse-variance weighted (IVW) method, complemented by MR-Egger, weighted median, and weighted mode approaches. Results were considered significant when IVW yielded *P* < 0.05 and directional consistency was observed between IVW and MR-Egger estimates in both directions. Significant findings were reported as odds ratios (ORs) with 95% confidence intervals (CIs). Reverse MR analyses were additionally conducted, in which SNPs significantly associated with gut microbiota were used as IVs to assess causal effects on dysmenorrhea.

#### 2.4.2 Sensitivity analysis

To ensure the validity of the instrumental variables, multiple sensitivity analyses were conducted. Horizontal pleiotropy was assessed using the MR-Egger intercept test (Burgess and Thompson, 2017), while heterogeneity across genetic instruments was examined using Cochran’s Q statistic (Cohen et al., 2015). A leave-one-out (LOO) analysis was further applied to identify potential influences of individual SNPs on the overall causal estimates.

All analyses were carried out using R software (version 4.4.3).

### 2.5 Animals, feeding, and treatment

Animal experiments received approval from the Experimental Animal Ethics Committee of Nanjing University of Chinese Medicine (No. ACU211205) and were conducted following the ARRIVE guidelines (Supplementary Data Sheet 1; Percie du Sert et al., 2020). A total of 20 female Sprague-Dawley (SD) rats, aged between 6 and 8 weeks and weighing 160 ± 20 g [license No. SCXK (SU) 2020-0009], were sourced from Beijing Vital River Laboratory Animal Technology Co., Ltd. (China). The rats were housed in specific pathogen-free (SPF) conditions (temperature 19 °C∼25 °C, 12 h light/dark cycle, relative humidity 40%–70%) with free access to food and water.

### 2.6 Primary dysmenorrhea (PDM) rat modeling procedures

All animals were acclimatized under standard laboratory conditions for 7 days before experimentation. Estrous cycles were monitored by vaginal smear, and only rats with regular 4-day cycles were included. Animals were randomly assigned (*n* = 5 per group) into four groups: control, PDM model, ibuprofen, and WJZTD. The chronic primary dysmenorrhea (PDM) model was induced using estradiol benzoate followed by oxytocin as previously described (Chinese Society of Traditional Chinese Medicine Experimental Pharmacology Professional Committee., 2018; Qin Y. et al., 2025). Briefly, rats in the model, ibuprofen, and WJZTD groups received repeated subcutaneous injections of estradiol benzoate followed by intraperitoneal oxytocin (OT) to induce dysmenorrhea, while the control group received saline at corresponding time points (Figure 1B).

From day 13, the ibuprofen and WJZTD groups received daily intragastric administration of ibuprofen (0.07 g⋅kg^–1^) or WJZTD (12.15 g⋅kg^–1^), respectively, calculated according to the human-to-rat body surface area conversion formula. The control and PDM groups received an equivalent volume of saline. On day 24, following the final induction of dysmenorrhea, rats were anesthetized with pentobarbital sodium (3%, 45 mg⋅kg^–1^, intraperitoneally). Blood was collected from the abdominal aorta, and rats were sacrificed by exsanguination. Serum, uterine tissues, and intestinal contents were immediately harvested, snap-frozen in liquid nitrogen, and stored at −80 °C until further analysis.

### 2.7 Writhing response assessment

Rats were habituated to the testing environment for at least 1 h before assessment. Following intraperitoneal oxytocin injection, the latency to the first writhing episode and the cumulative writhing score within a 30-min observation period were recorded as previously described (Qin Y. et al., 2025). The writhing behavior was assessed on a scale of 0–3, as described by Schmauss and Yaksh (1984) in the Supplementary Table 2, and all assessments were performed by investigators blinded to group allocation.

### 2.8 Enzyme-linked immunosorbent assay (ELISA)

Serum PGF2α concentrations were determined using a commercial ELISA kit (AF9456-A, AiFang Biological, China) according to the manufacturer’s instructions.

### 2.9 Hematoxylin-eosin (HE) staining

Uterine tissues were fixed in 4% paraformaldehyde for over 24 h, embedded in paraffin, and sectioned at 5 μm thickness. Hematoxylin and eosin staining (Sigma, USA, Batch No. H9627 and 170) was performed as previously described (Maehara and Fujimori, 2021), and histological observations were made using a light microscope (Nikon Eclipse E100, Tokyo, Japan).

### 2.10 Extraction of fecal DNA and 16S rRNA analysis

Total DNA was extracted from intestinal contents and quantified with a NanoDrop 2000 spectrophotometer (Thermo Fisher Scientific, USA), while integrity was verified by 1.2% agarose gel electrophoresis. The V3–V4 hypervariable regions of the bacterial 16S rRNA gene were amplified using primers 341F (5′-ACTCCTACGGGAGGCAGCA-3′) and 806R (5′-GGACTACHVGGGTWTCTAAT-3′) (Claesson et al., 2009), with ultrapure water serving as a negative control to avoid false-positive amplification. PCR products were subsequently purified with VAHTS DNA Clean Beads at a bead-to-sample ratio of 0.8× (Vazyme, China), quantified using Quant-iT PicoGreen dsDNA Assay Kit (Thermo Fisher Scientific, USA), and applied for sequencing library preparation on the Illumina TruSeq platform (Illumina, USA).

Raw sequences were first truncated to remove barcode and primer regions, followed by quality filtering and chimera removal using Vsearch (v2.3.4) with minor parameter adjustments. The resulting feature table was rarefied to the same sequencing depth across samples by random subsampling, and Amplicon Sequence Variants (ASVs) or Operational Taxonomic Units (OTUs) were obtained. Based on their distribution among samples, microbial diversity was further assessed.

Alpha diversity and beta diversity were both calculated using QIIME2. Alpha diversity was assessed using seven indices (Chao1, Observed species, Good’s coverage, Shannon, Simpson, Faith’s PD, and Pielou’s evenness), while beta diversity was computed and subsequently visualized using R software (version 4.4.3). Taxonomic differences between groups were identified by LEfSe analysis. Functional prediction was performed using PICRUSt2, and the resulting functional profiles were annotated based on the MetaCyc database^2^ (Segata et al., 2011). Other plots and statistical analyses were performed with the R package.

### 2.11 Correlation analyses

Correlation analyses were performed to assess associations between gut microbiota and pain-related parameters, including PG levels, writhing score, and writhing latency on day 24, with significance defined as *P* < 0.05 and |Spearman’s ρ| > 0.4, and where applicable, false discovery rate (FDR) adjustment was applied. Potential therapeutic differences between the ibuprofen and WJZTD were examined by identifying bacterial genera that exhibited distinct correlation patterns with the same pain parameter in both groups. All statistical analyses and visualizations were conducted in R.

### 2.12 Statistical analysis for *in vivo* experiments

Data analysis and visualization were performed using GraphPad Prism 8.0.1 software. Differences between groups were evaluated by one-way or two-way analysis of variance (ANOVA), with statistical significance set at *P* < 0.05. Results are expressed as the mean ± standard deviation (SD), and specific statistical methods are described in the corresponding figure legends.

## 3 Results

### 3.1 MR analyses identify the causal association between dysmenorrhea and gut microbiota

Genetic instruments for gut microbiota were extracted at phylum, class, order, family, and genus levels, with F-statistics exceeding 10 for all IVs, indicating that weak instrument bias is unlikely (Supplementary Table 3, corresponding to original Supplementary Table 1).

As shown in Figure 1C, genetic liability to seven gut microbiota taxa (one class, two orders, one family, and three genera) was associated with dysmenorrhea based on IVW estimates. With MR-Egger, Weighted Mode, and Weighted Median analyses performed for comparison (Supplementary Table 3, corresponding to original Supplementary Tables 2, 3). Dysmenorrhea had a positive causal effect on the class *Erysipelotrichia*, its subordinate order *Erysipelotrichales*, and family *Erysipelotrichaceae* (OR = 1.052, 95% CI = 1.004∼1.102, *P* = 0.034), reflecting their taxonomic hierarchy, as well as the independent order Mollicutes RF9 (OR = 1.080, 95% CI = 1.015∼1.149, *P* = 0.015). In contrast, dysmenorrhea was negatively associated with the genera *Eubacterium xylanophilum group* (OR = 0.922, 95% CI = 0.870∼0.978, *P* = 0.006), *Lachnospiraceae NK4A136 group* (OR = 0.952, 95% CI = 0.907∼0.999, *P* = 0.045), and *Roseburia* (OR = 0.932, 95% CI = 0.889∼0.977, *P* = 0.003). Notably, the three genera belong to the same family, Lachnospiraceae, which comprises members that can degrade polysaccharides and produce short-chain fatty acids, particularly butyrate, a protective factor against inflammation and pain.

Effect directions and magnitudes were largely consistent between IVW and Weighted Median estimates, supporting the robustness of the results. MR-Egger regression intercepts indicated no evidence of directional pleiotropy, Cochran’s *Q*-tests showed no significant heterogeneity (*P* > 0.05, Supplementary Table 3, corresponding to original Supplementary Tables 4, 5), and leave-one-out analyses at all taxonomic levels confirmed the reliability of the findings.

### 3.2 Reverse MR validation for causal directionality

In the reverse MR analyses, we further evaluated the causal directionality between gut microbiota and dysmenorrhea. All taxa included in the reverse analysis were strongly associated with dysmenorrhea (F-statistic > 10), and four MR methods were applied consistently with the forward analysis. For phylum Euryarchaeota (id.55), weak but nominally significant associations with dysmenorrhea were observed in the IVW analysis (β = −0.409; 95% CI: −0.793∼−0.025; *P* = 0.036), with directionally consistent estimates across IVW, MR-Egger, and Weighted Median. No evidence of heterogeneity (Q_pval = 0.542) or horizontal pleiotropy (intercept *P* = 0.568) was detected, and leave-one-out analysis identified no outlier SNPs (Supplementary Data Sheet 2). However, this finding was based on only three SNPs, indicating limited statistical power and insufficient evidence to support a causal effect of gut microbiota on dysmenorrhea (Supplementary Table 4).

### 3.3 Pharmacotherapy alleviates the pain and modulates the composition of gut microbiota in PDM rats

**FIGURE 2:**
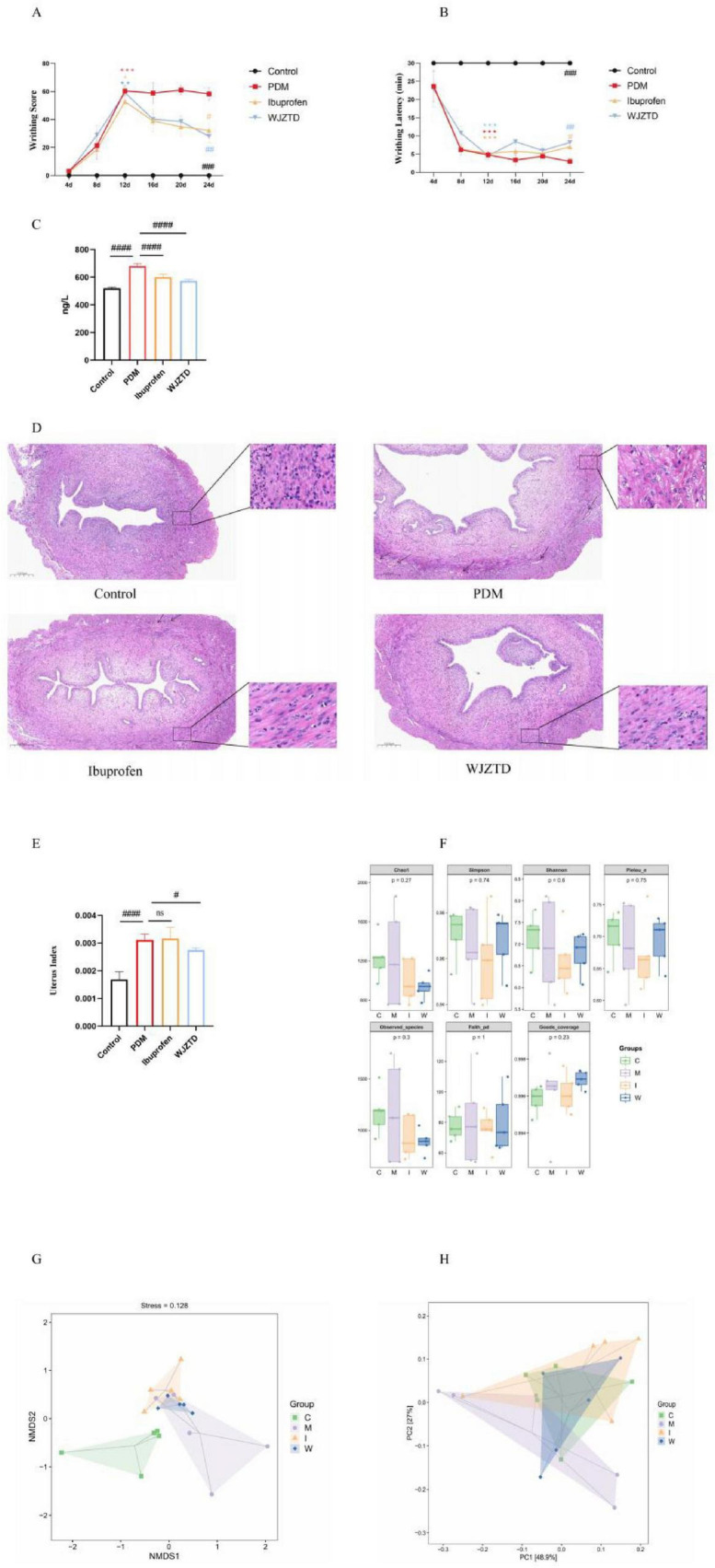
**(A,B)** The writhing scores and writhing latency of each group(*n* = 5). **(C)** Serum PGF2α level (*n* = 5). **(D)** Uterine tissue hematoxylin-eosin (H&E) staining in rats (200×), the congested uterine spiral artery is indicated by an arrow. Scale bars, 312.5 μm. **(E)** Uterine organ index of each group (*n=5*). Compared with the control group, *P < 0.05, **P < 0.01, ***P < 0.001, ****P < 0.001. Compared with the model group, ns, no significant, ^#^*P* < 0.05, *^##^P* < 0.01, *^###^P* < 0.001, *^####^P* < 0.001. **(F)** α-diversity of the gut microbiota at the OTU level. **(G,H)** NMDS and PCA plots showing β-diversity at the OTU level.

To evaluate the establishment of the PDM model and the therapeutic effects of drug intervention, serum PGF2α levels, writhing score and latency, and uterine organ index were measured (Hong et al., 2022). Following the third OT injection on day 12, the PDM model (M), ibuprofen (I), WJZTD (W, TCM), and control (C) groups were compared. The PDM model, and drug-invention groups showed significantly increased writhing scores and shortened latency compared to controls (writhing score: M: *P* < 0.001, I: *P* < 0.01, W: *P* < 0.05; writhing latency: *P* < 0.001 for all comparisons), confirming successful modeling, with no differences in body weight observed among groups (Figures 2A, B and Supplementary Image 1). After 12 days of treatment, both drug intervention groups exhibited marked improvements in writhing responses (writhing score: C: *P* < 0.001, I: *P* < 0.01, W: *P* < 0.05; writhing latency: C: *P* < 0.001, I: *P* < 0.01, W: *P* < 0.05) and reductions in serum PGF2α levels compared with the model group (C: *P* < 0.001, I: *P* < 0.001, W: *P* < 0.001) (Figure 2C). As demonstrated in our previous study (Yang X. et al., 2024), HE staining of uterine tissue revealed severe uterine pathology in the PDM group, including disrupted smooth muscle cells, disorganized fibers, and spiral artery congestion, which was partially restored by drug intervention (Figure 2D). However, significant differences in the uterine organ index relative to the model group were observed only in the control and TCM-treated groups, whereas no change was detected in the ibuprofen group (Figure 2E).

To characterize the alterations in gut microbiota associated with dysmenorrhea and to explore the mechanisms of drug intervention, 16S rRNA gene sequencing was performed on fecal samples from the four groups. Analysis of α-diversity indices, reflecting microbial richness and diversity, revealed no significant differences among groups (Figure 2F and Supplementary Table 5, corresponding to original Supplementary Table 1). Nevertheless, β-diversity analysis demonstrated clear compositional variations. NMDS (Stress = 0.128) and PCA (PC1 = 48.9%; PC2 = 27.0%) analysis both indicated significant separation in microbial community structure across the four groups (Figures 2G, H and Supplementary Table 5, corresponding to original Supplementary Tables 2, 3). Furthermore, PCA analysis showed that the microbiota profiles of drug-treated groups, particularly the TCM-treated group, clustered closer to the controls but remained distinct from the PDM group. These findings suggest that dysmenorrhea primarily alters microbial composition rather than overall richness or diversity.

### 3.4 Identification of signature taxa in intestinal microbiota and functional alterations prediction

Given that the analgesic effects of drug intervention may be associated with changes in gut microbiota, we selected the top 20 abundant taxa across multiple taxonomic levels, with the main text focusing on the family and genus levels.

**FIGURE 3:**
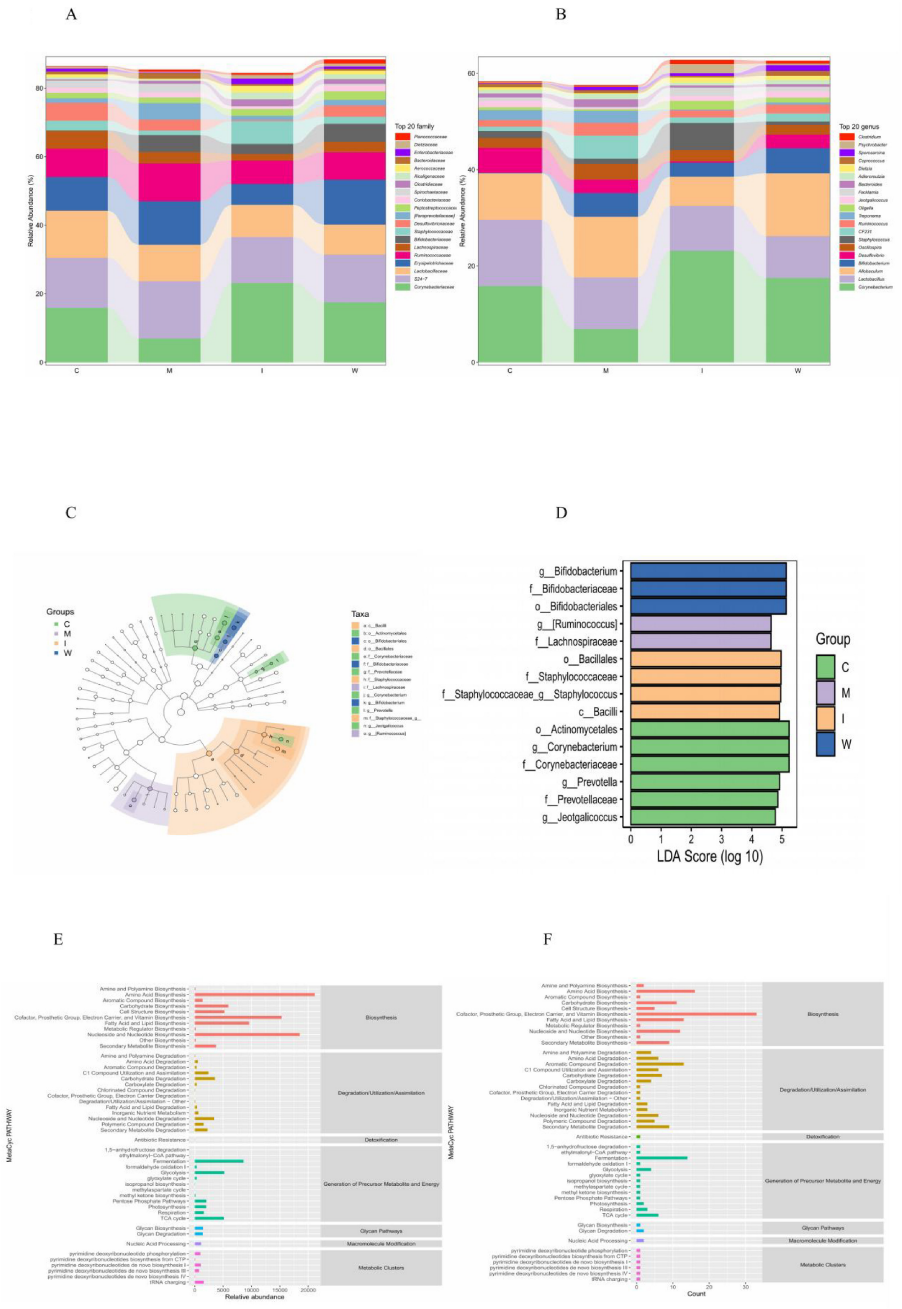
**(A)** Top 20 relative abundances of gut microbiota at the family level. **(B)** Top 20 relative abundances of gut microbiota at the genus level. **(C,D)** Distribution of differential taxa identified by LEfSe analysis. **(E)** Prediction of functional pathways using PICRUSt2 based on the relative abundance of gut microbiota. **(F)** Prediction of functional pathways using PICRUSt2 based on the occurrence frequency of gut microbiota.

At the family and genus levels (Figures 3A, B and Supplementary Table 6, corresponding to original Supplementary Tables 1, 2), the PDM model group (M) exhibited pronounced dysbiosis compared with the control group (C). Corynebacteriaceae and Lachnospiraceae were markedly reduced, whereas Erysipelotrichaceae, Ruminococcaceae, and Paraprevotellaceae were elevated. These alterations were largely reversed by drug treatment. Within the intervention groups, the TCM-treated group (W) displayed an increase in Bifidobacteriaceae, whereas the ibuprofen group (I) was characterized by elevated Corynebacteriaceae and Staphylococcaceae together with reduced Bifidobacteriaceae. At the genus level, compared with the control group, the model group showed increased *CF231* (f_Paraprevotellaceae) and decreased *Corynebacterium* (f_Corynebacteriaceae), consistent with the family-level changes. Compared with the model group,both intervention strategies modulated this dysbiotic state: the ibuprofen group exhibited a decrease in *Allobaculum* (f_Erysipelotrichaceae) and an increase in *Staphylococcus* (f_Staphylococcaceae), whereas the TCM-treated group showed a distinct increase in *Bifidobacterium*. Notably, Erysipelotrichaceae and its genus *Allobaculum* remained consistently enriched in both the M and W groups, exhibiting a uniform increasing trend across multiple taxonomic levels.

To further identify taxa significantly associated with dysmenorrhea, LEfSe analysis was performed, with Linear Discriminant Analysis (LDA) scores > 2.0 considered significant. The most differentially abundant taxa varied among the groups: *Ruminococcus* (f_Lachnospiraceae) contributed most in the model group (M), *Staphylococcus* (f_Staphylococcaceae) in the ibuprofen-treated group (I), and *Bifidobacterium* (f_Bifidobacteriaceae) in the TCM-treated group (W), whereas *Corynebacterium* (f_Corynebacteriaceae), *Prevotella* (f_Prevotellaceae), and *Jeotgalicoccus* were predominant in the control group (C) (Figures 3C, D and Supplementary Table 6, corresponding to original Supplementary Table 3). These findings indicate that each intervention was associated with distinct alterations in gut microbiota linked to dysmenorrhea, the implications of which warrant further investigation.

As shown in Figures 3E, F (Supplementary Table 6, corresponding to original Supplementary Tables 4, 5), functional prediction analysis revealed that highly abundant and broadly distributed pathways mainly involved amino acid metabolism, encompassing both biosynthesis and degradation, as well as nucleoside and nucleotide biosynthesis, which are core functions essential for host–microbe homeostasis. Beyond these fundamental processes, enrichment of aromatic compound degradation pathways was particularly noteworthy. By comparing differential pathways between the C and M groups, and between the W and M groups (*P* < 0.05), and focusing on those with opposite log fold-change (FC) trends, we identified three candidate pathways potentially influenced by TCM treatment: PWY0-321 (phenylacetate degradation I), PWY-5181 (toluene degradation III, via p-cresol), and PWY-6185 (4-methylcatechol degradation) (Supplementary Table 6, corresponding to original Supplementary Tables 6, 7). All are closely linked to microbial catabolism of aromatic amino acid–derived metabolites, suggesting that aberrant regulation of these pathways may contribute to dysmenorrhea, while TCM intervention may help modulate these alterations.

### 3.5 Correlation analysis of gut microbiota with pain indicators and differential modulation by pharmacological interventions

**FIGURE 4:**
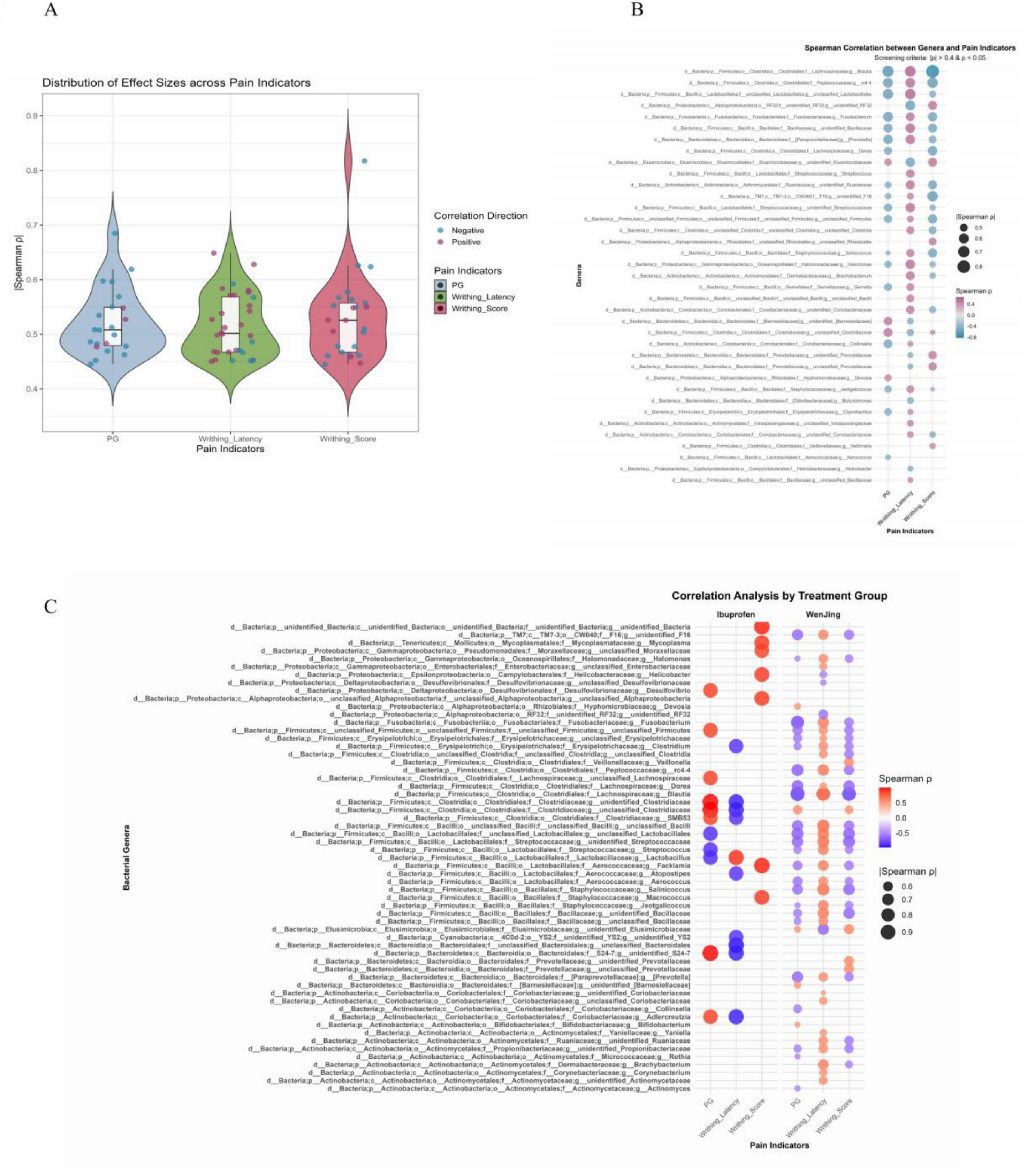
**(A)** Overall effect size distribution of correlations between gut microbiota and pain-related parameters. **(B)** Correlations between individual gut microbial taxa and pain-related parameters. **(C)** Correlation analysis by treatment group.

Correlation analysis revealed widespread associations between gut microbiota and pain-related indicators, with most correlations showing moderate to strong effect sizes (|ρ| > 0.4) and many exceeding 0.6 (Figures 4A, B and Supplementary Table 7, corresponding to original Supplementary Table 1), highlighting the biological relevance of microbial alterations in dysmenorrhea-associated phenotypes. Among the genera examined, *Blautia* (f_Lachnospiraceae) was the only genus significant for Writhing Score (FDR < 0.05), showing a negative correlation with serum PG level and a positive correlation with Writhing Latency, reflecting a balanced pain-modulating pattern. These trends align with MR and *in vivo* observations at the family level, suggesting a protective role.

When stratified by treatment, ibuprofen and TCM-treated groups exhibited distinct microbiota–pain correlation patterns (Figure 4C and Supplementary Table 7, corresponding to original Supplementary Table 2). In the ibuprofen group, 28 significant correlations were observed, 11 of which involved PG index, supporting its focused effect on prostaglandin-mediated pathways. In contrast, the TCM group showed 91 significant correlations, consistent with broader modulation of pain-related microbiota, in line with the multi-target therapeutic mechanism of TCM, which may involve regulation of specific gut microbes, including *Blautia*.

## 4 Discussion

In this study, we integrated bidirectional MR analyses with *in vivo* experiments to investigate the causal relationship between dysmenorrhea and gut microbiota. Our findings provide convergent genetic and experimental evidence that dysmenorrhea causally alters specific microbial taxa, particularly members of the Lachnospiraceae and Erysipelotrichaceae families. Importantly, these results highlight that dysmenorrhea-induced microbial dysbiosis represents a downstream pathological state that can be therapeutically targeted: restoring beneficial taxa such as *Blautia* and *Bifidobacterium* may modulate pain and alleviate symptoms, underscoring gut microbiota modulation as a potential strategy for dysmenorrhea management.

### 4.1 MR highlights the causal direction from dysmenorrhea to gut microbiota

The forward MR analysis revealed that genetic liability to dysmenorrhea was associated with a reduced abundance of three genera within the Lachnospiraceae family, which has been widely implicated in pain regulation through both metabolic and immune-mediated pathways (Shi et al., 2025; Zhao et al., 2024). Among its metabolites, butyrate plays a pivotal role and has been shown to markedly alleviate pain hypersensitivity in Parkinson’s disease (PD) mouse models (Avagliano et al., 2025). Clinical evidence further supports this mechanism, as elevated circulating butyrate levels following FMT are associated with pain improvement (Bonomo et al., 2020). Consistently, downregulation of the Lachnospiraceae family was also observed in the PDM rat model.

By contrast, the reverse MR analysis provided no robust evidence that gut microbiota causally influence dysmenorrhea, aside from a weak nominal association with Euryarchaeota based on only three SNPs. This reinforces the interpretation that gut microbial changes are largely a consequence of dysmenorrhea rather than its cause. Nevertheless, these findings may have been affected by limited statistical power, reliance on SNPs obtained exclusively from East Asian datasets, and the categories of dysmenorrhea, which can be classified into primary and secondary, the latter arising from pathological changes in the pelvic organs, such as adenomyosis and endometriosis. Future MR analyses involving larger, multi-ethnic cohorts and stratification by dysmenorrhea subtype will be essential to determine whether specific microbial taxa exert causal effects on disease risk.

### 4.2 Gut microbiota profiles in experimental treatment groups

To validate the MR findings, we employed a PDM rat model, which has been extensively characterized in our previous work. For pharmacological interventions, we selected the frontline treatment for dysmenorrhea, the NSAID ibuprofen, and a traditional Chinese medicine, WJZTD, which we had previously demonstrated to be effective. These interventions produced distinct microbial signatures. In the ibuprofen group, *Staphylococcus* (f_Staphylococcaceae) was notably enriched, consistent with previous reports that NSAIDs can increase the abundance of Gram-positive bacteria (Hodkovicova et al., 2022). This enrichment may be related to ibuprofen-induced reactive oxygen species (ROS) production and alterations in bile acid metabolism (Zeng et al., 2022), and is more likely associated with NSAID-induced side effects, such as mucosal injury and dysbiosis, rather than with the therapeutic mechanisms of the drug (Lee et al., 2025).

Wenjing Zhitong Decoction treatment, on the other hand, increased the abundance of *Bifidobacterium* (f__Bifidobacteriaceae), a genus that has been reported to be significantly reduced in the gut microbiota of patients with dysmenorrhea (Nie et al., 2024). *Bifidobacterium* exerts anti-inflammatory effects by modulating the gut microbial community and is also involved in tryptophan metabolism, the precursor of serotonin 5-hydroxytryptamine, which plays a key role in pain modulation (Qin W. et al., 2025). This finding aligns well with our functional prediction, which indicated that WJZTD modulates microbial pathways involved in aromatic amino acid catabolism. *Bifidobacterium* intervention has been reported to significantly alleviate pain in patients with fibromyalgia (Cai et al., 2025). Conversely, a reduction in *Bifidobacterium* may contribute to neurotransmitter imbalance and exacerbate pain perception (Galley et al., 2023).

### 4.3 Pharmacological interventions alter β-diversity rather than α-diversity

Both ibuprofen and WJZTD exerted significant analgesic effects in the PDM rat model. Interestingly, drug interventions primarily influenced β-diversity, reflecting alterations in microbial community composition, whereas α-diversity indices remained unchanged. This supports the concept that targeted modulation of specific taxa, rather than global microbiota diversity, underlies therapeutic effects in dysmenorrhea (Kashyap and Wang, 2024; Minerbi and Shen, 2022).

### 4.4 *Blautia* as a protective taxon in pain regulation

Correlation analyses demonstrated that *Blautia* was negatively associated with pain indicators in the WJZTD group. Of note, *Blautia* (f_Lachnospiraceae) was the only genus that remained significant after FDR correction. *Blautia* has been implicated in pain regulation through multiple pathways. Reduced *Blautia* abundance has been linked to a higher risk of neuropathic pain in hematopoietic cell transplant recipients (Rashidi et al., 2022), whereas elevated *Blautia* levels have been significantly associated with symptom relief in patients treated with linaclotide for constipation and abdominal pain (Zhou et al., 2024). Collectively, these findings highlight the restoration of *Blautia* abundance as a potential strategy for pain management. Notably, based on Spearman correlations, *Blautia* exhibited a protective pattern only in the WJZTD group, whereas ibuprofen showed a disrupted profile, suggesting that its beneficial role is more likely mediated by WJZTD.

Additionally, we also observed alterations in members of the Erysipelotrichaceae family. Consistent with our MR analyses, Erysipelotrichaceae abundance was increased in the model group, supporting a potential pathogenic role in PDM. This is in line with previous evidence showing that FMT from fibromyalgia patients enriched in Erysipelotrichaceae into germ-free mice can induce pain-like behaviors (Cai et al., 2025). At the genus level, *Allobaculum* (f_Erysipelotrichaceae) was elevated in both the model and WJZTD groups, while correlation analyses revealed that *Coprobacillus* (f_Erysipelotrichaceae) was positively associated with serum PG levels and writhing scores but negatively associated with latency, further implicating this family in pain regulation. Unexpectedly, Erysipelotrichaceae abundance was also increased following WJZTD treatment, whereas it was reduced in the ibuprofen group. The biological significance of this discrepancy remains unclear. One possibility is that the elevation reflects a compensatory microbial shift driven by the multi-component nature of WJZTD, rather than a direct pathogenic effect.

Given the complex, multi-component nature of WJZTD, its modulatory effects on the gut microbiota are likely driven by specific herbal constituents. Several herbal components of WJZTD, which have been well-characterized in our previous studies (Yang X. et al., 2024), may contribute to the observed microbial modulation. For instance, volatile compounds from *Artemisia argyi* Levl.Et Vant. (Asteraceae; ARTEMISIAE ARGYi FOLIUM) have been reported to enrich Lachnospiraceae (including *Blautia*), thereby ameliorating dysbiosis (Zhou et al., 2025), while polysaccharides in *Dioscorea opposita* Tunb. (Dioscoreaceae; DIOSCOREAE RHIZOMA) may act as prebiotics to promote *Blautia* growth (Yang et al., 2022). Moreover, the high dietary fiber content of *D. opposita* Tunb. is known to facilitate the proliferation of *Bifidobacterium* (Wang et al., 2022). In contrast, ibuprofen appears to exert no direct regulatory effect on *Blautia* (Oberman et al., 2021).

### 4.5 Limitations and future directions

This study has several limitations. First and most notably, the generalizability of our Mendelian randomization findings is constrained by the exclusive use of GWAS summary statistics from East Asian populations. We explicitly acknowledge this as a primary limitation, as it may restrict the extrapolation of the causal inferences to other ethnic groups. Further studies utilizing larger, multi-ethnic cohorts are essential to validate and extend these results. Second, the sample size in our animal experiment (*n* = 5 per group), while appropriate for a preliminary exploratory study, is relatively small and may limit the statistical power of the findings. Future studies with larger sample sizes are warranted to confirm these results. Third, only a PDM model was used for animal validation, and the absence of secondary dysmenorrhea models restricts extrapolation. Fourth, functional predictions were inferred using PICRUSt2, which requires further validation through metagenomic or metabolomic analyses. Finally, to avoid overlooking potentially meaningful associations, we also examined correlations with |ρ| > 0.4 and nominally significant *P*-values, even if they did not reach FDR significance. However, due to the risk of false positives, these findings should be further validated.

## 5 Conclusion

This study demonstrates that dysmenorrhea causally alters gut microbiota composition, particularly reducing Lachnospiraceae and increasing Erysipelotrichaceae. In a PDM rat model, both ibuprofen and WJZTD alleviated pain but produced distinct microbial signatures. *Blautia* was identified as a key genus whose restoration correlates with pain reduction, highlighting its potential as a therapeutic target. Overall, our findings underscore the importance of gut microbiota modulation in dysmenorrhea management and provide a foundation for microbiota-targeted interventions. Future studies with multi-ethnic cohorts, secondary dysmenorrhea models, and functional validation are warranted to further elucidate these mechanisms.

## Data Availability

The GWAS summary statistics for dysmenorrhea are available from the IEU Open GWAS project under accession code ebi-a-GCST006636. The gut microbiota GWAS summary data were obtained from the study by Kurilshikov et al. (doi: 10.1038/s41588-021-00913-z). All other data supporting the findings of this study, including all numerical data points used to generate graphs and perform statistical analyses, are available within the article, its Supplementary material, or from the corresponding author upon reasonable request.

## Footnotes

1
https://gwas.mrcieu.ac.uk/

2
https://metacyc.org/
