# Supplementary material for: Gut microbiota in dysmenorrhea: causal evidence from Mendelian randomization and microbial-targeted intervention validation
Source: Front Microbiol. 2026 Feb 2;16:1720643. doi: 10.3389/fmicb.2025.1720643 (PMC12907366; doi:10.3389/fmicb.2025.1720643)
Supplement: Supplementary file 2 [file Table_2.docx]

# Table1 Scale of writhing response in dysmenorrhea rats

| **Level** | **Behavioral performance** | **Score/point** |
| --- | --- | --- |
| 0 | Normal posture (paw placed flat at the bottom of the box or normal probing behavior) | 0 |
| 1 | Body tilted to one side | 1 |
| 2 | Hind limb extension, hind paw dorsiflexion, body extension with frequent lateral pelvic rotation | 2 |
| 3 | Abdominal muscle contraction and hind limb backward extension | 3 |
