# Supplementary material for: Gut microbiota in dysmenorrhea: causal evidence from Mendelian randomization and microbial-targeted intervention validation
Source: Front Microbiol. 2026 Feb 2;16:1720643. doi: 10.3389/fmicb.2025.1720643 (PMC12907366; doi:10.3389/fmicb.2025.1720643)
Supplement: Supplementary file 9 [file Data_Sheet_2.pdf]

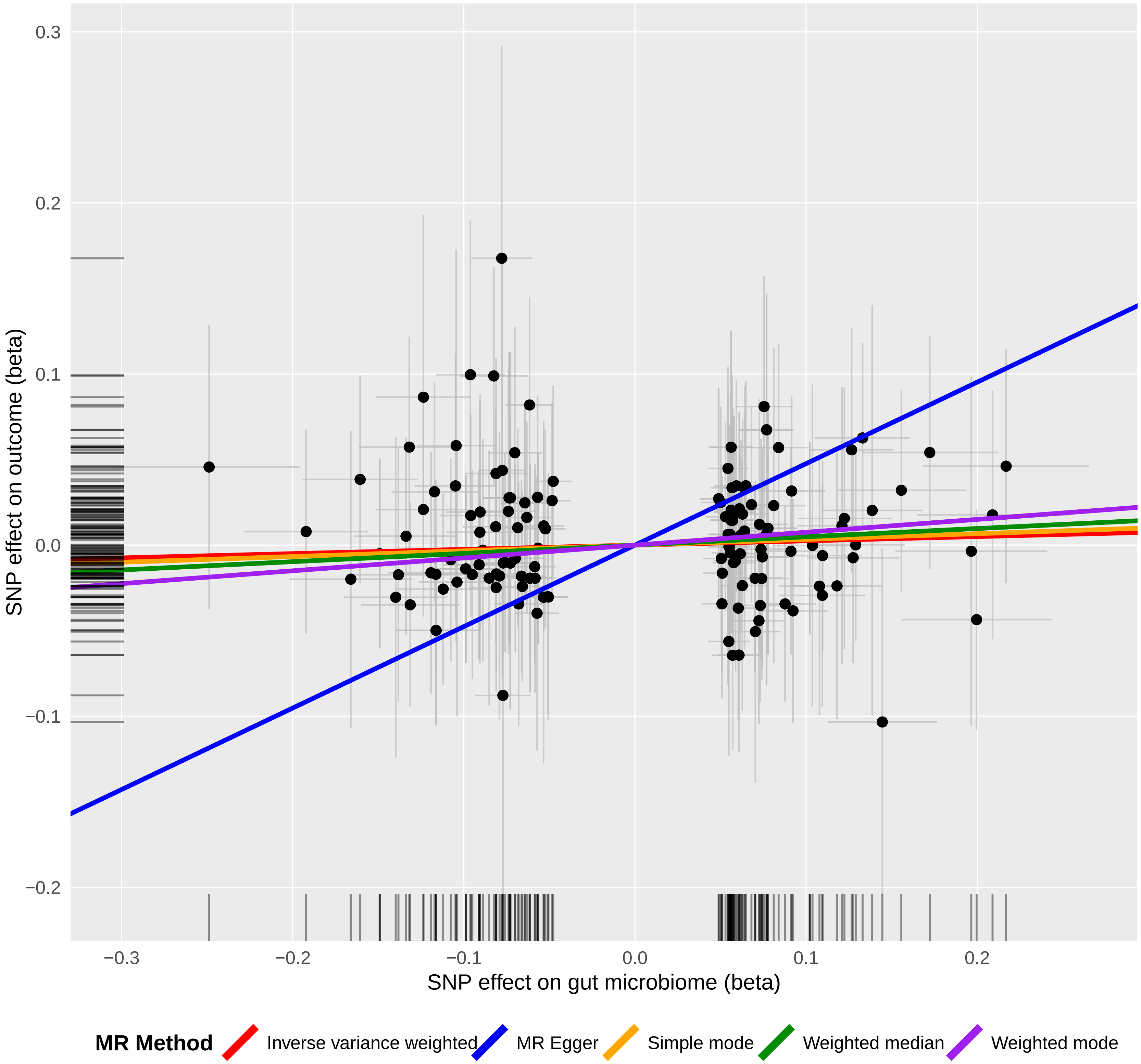
0.3
0.2
0.1
0.0
−0.1
−0.2
SNP effect on outcome (beta)
−0.3
−0.2
−0.1
0.0
0.1
0.2
SNP effect on gut microbiome (beta)
MR Method
Inverse variance weighted
MR Egger
Simple mode
Weighted median
Weighted mode

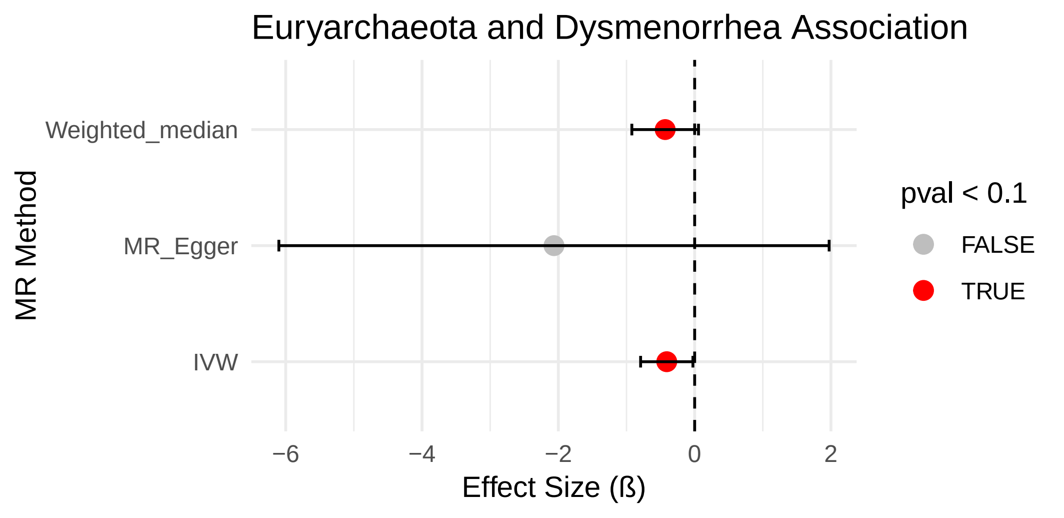

Euryarchaeota and Dysmenorrhea Association
Weighted_median
MR_Egger
IVW
MR Method
−6
−4
−2
0
2
Effect Size (ß)
pval < 0.1
FALSE
TRUE

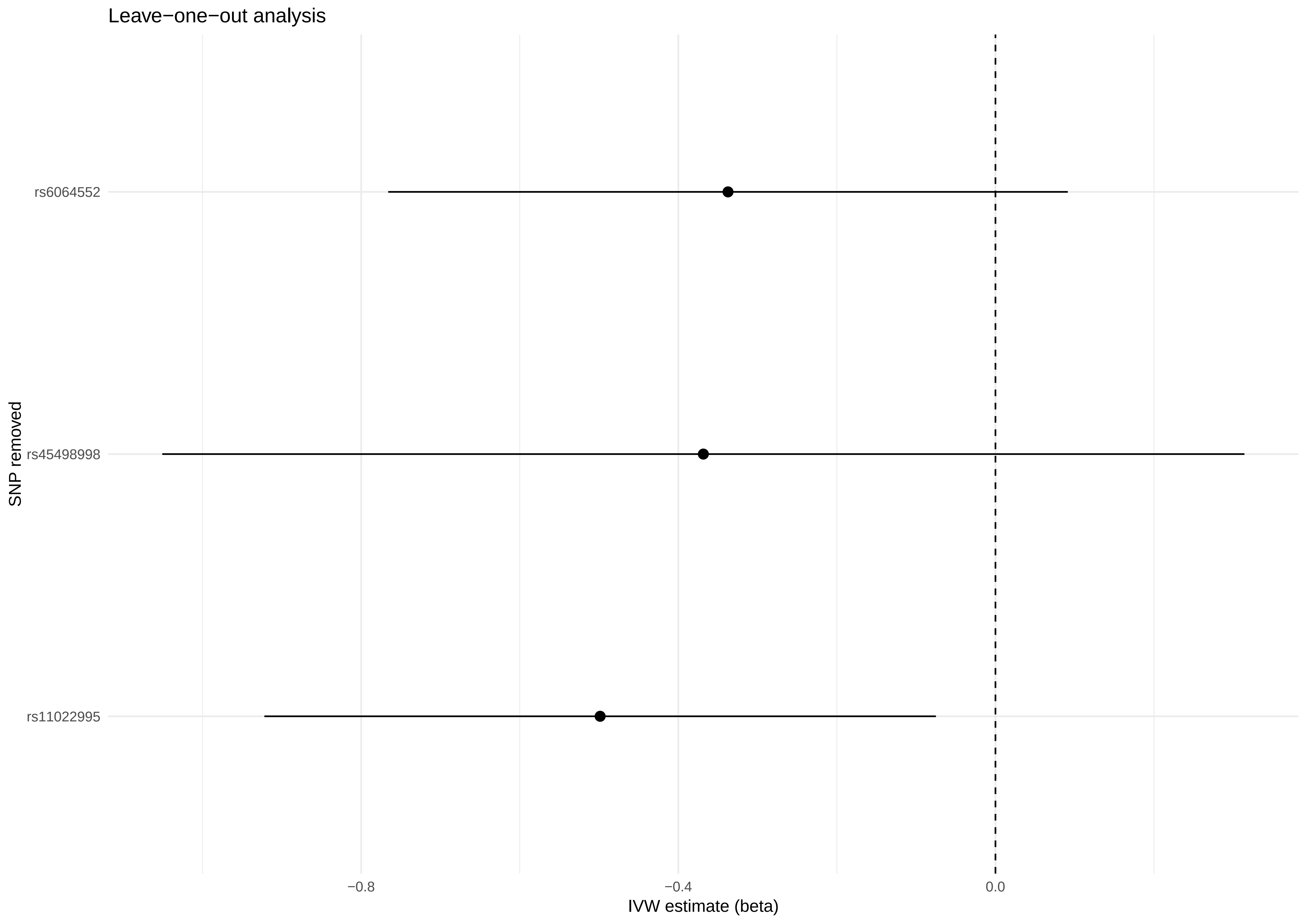
Leave−one−out analysis
rs6064552
rs45498998
rs11022995
SNP removed
−0.8
−0.4
0.0
IVW estimate (beta)
