# Supplementary figures and images for: Gut microbiota in dysmenorrhea: causal evidence from Mendelian randomization and microbial-targeted intervention validation
Source: Front Microbiol. 2026 Feb 2;16:1720643. doi: 10.3389/fmicb.2025.1720643 (PMC12907366; doi:10.3389/fmicb.2025.1720643)

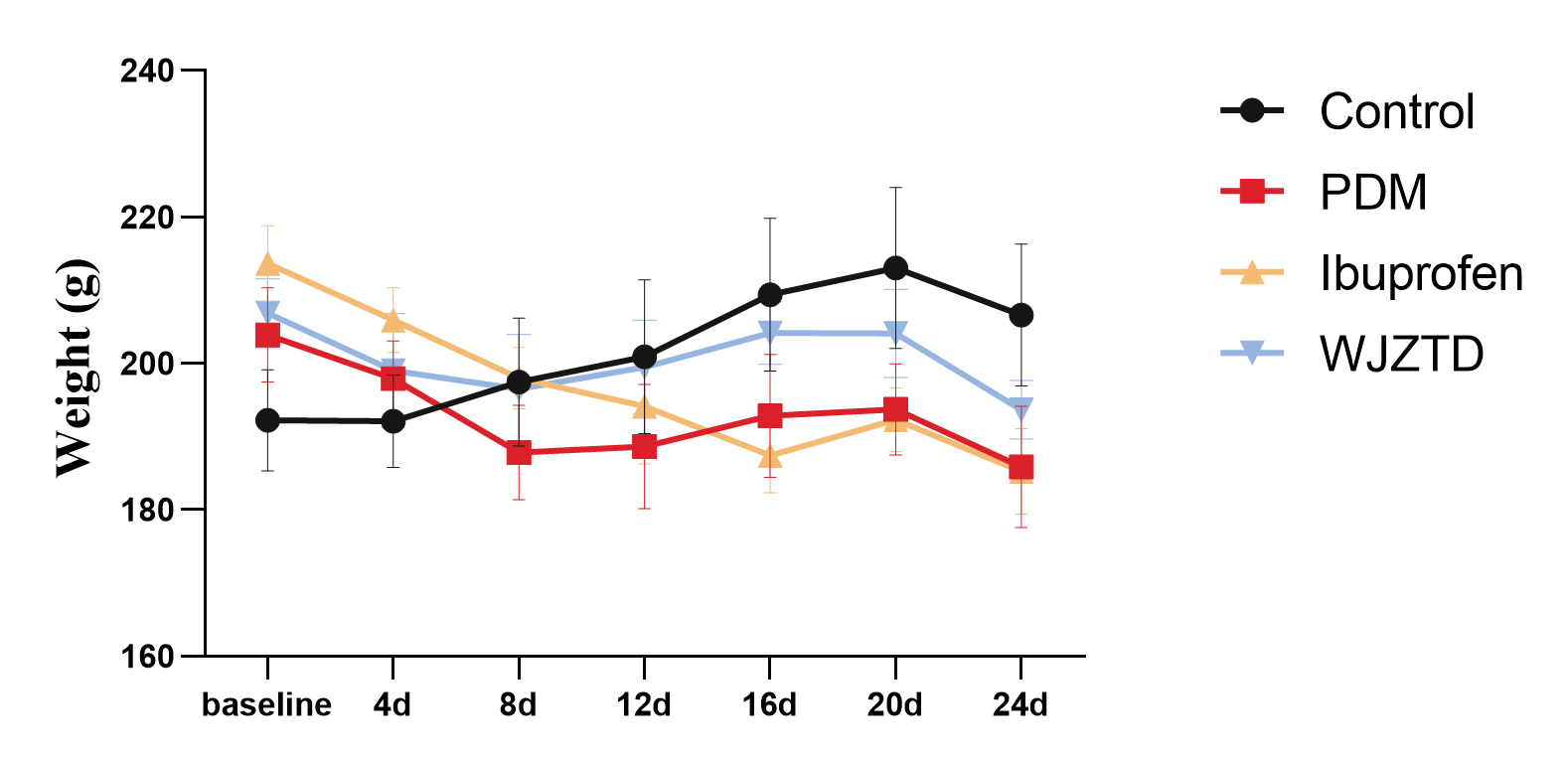

Supplement: Supplementary file 10 [file Image_1.tif]
